# Hints and Improvements in the Practice of Medicine, Surgery, and Pharmacy

**Published:** 1800-04

**Authors:** 


### HINTS AND IMPROVEMENTS

### IN THE PRACTICE OF

### MEDICINE, SURGERY, AND PHARMACY.

---

### *On the Medicinal Effects of the Salt of Tartar in Puerperal Fevers, &c.*

[ Concluded from our last Number, pp 264—267. ]

OBSERVATION VI.

THE female Cit. Vitry, twenty-eight years of age, of a very strong and muscular constitution, experienced a violent fright on the fourth day after her delivery. An acute fever, attended with delirium, soon succeeded. The patient was greatly agitated, being tormented with the most distressing reveries. Her breasts sunk, the abdomen became distended and painful, and the lochia were suppressed. The only means employed were the application of a dozen leeches to the vagina, the carbonat of potass, some composing draughts, and after the sixth day, purgatives. These remedies speedily produced the best effects, and the cure was completed between the twentieth and twenty-fifth days.

OBSERVATION VII.

In the month of March, 1789, the female Cit. Parquin, twenty-six years of age, robust, and of a good constitution, met with great vexation immediately after her delivery. Cit. Guinot, who was called in on the fourth day, found the patient labouring under the following symptoms: her breasts were soft, the region of the uterus was painful and tense, irritation over the whole abdomen, suppression of the lochia, violent head-ach, attended with delirium, acute cough, harsh voice, sharp and quick speech, great difficulty of breathing, wild and sparkling eyes, inflamed face, and a small, quick, and contracted pulse. The application of leeches, the use of the carbonat of potass, topical applications to relax the abdomen, clysters containing a drachm of soap in each, the white electuary of the Pharmacopœa, with three drachms of pectoral syrup and twelve grains of carbonat of potass, an aperient and pectoral diet drink, with purgatives towards the termination of the paroxysm, gradually calmed all the above symptoms, and by the tenth tenth day a cure was effected. The carbonat of potafs was occafionally continued the fucceeding fortnight.

### Observation VIII. and Last.

In the year 1784, the young female Cit. Gey, of a ftrong conftitution, was, in confequence of a fudden alarm, attacked on the third day after her lying-in with an acute fever and an extraordinary head-ach. Her fkin was parched and burning; the tongue arid, fucceeded by frequent ftartings of the tendons; and fhe had an inceffant and convulfive cough. Her breafts were foft and wrinkled, the lochia fuppreffed; the abdomen, and particularly the uterine region, were much diftended and painful. Cit. Guinot prefcribed a decoction of barley with fyrup of violets and nitre, a white electuary, with two drachms of fyrup of poppies and ten grains of potafs, of which half a fpoonful was to be taken every half hour; and emollient fomentations and clyfters, of the fame ingredients, were ordered three times a day. He directed fifteen leeches to be applied, which produced a good effect. On the fifth day, the fymptoms had confiderably abated. The phyfician then prefcribed two clyfters daily, compofed of two drachms of bark and one drachm of the carbonat of foda. Befide thefe, he directed foap to be added to the emollient fomentations; and after the patient had been purged on the fourteenth day, fhe was fpeedily reftored to perfect health. She continued, however, to ufe the carbonat of potafs for a fortnight after, with a view to prevent a relapfe.

In 1790, the fame woman became fubject to a fimilar difeafe, and was treated in a fimilar manner. On the fixth day of the treatment, the fymptoms having almoft difappeared, fhe experienced a fudden change, which was attended with very painful ftitches in the fide, copious fpitting of blood, a violent cough, and an ardent fever. Two bleedings in the arm, pectoral drinks, the white electuary, with the fyrup of poppies and emollient clyfters, allayed this new attack in the fpace of ten days. At this period the patient was feized with a ftupifying delirium, and became fubject to paralyfis of the left fide. Cit. Guinot applied on the paralytic arm and thigh fome well camphorated blifters, which were dreffed with the ointment of ftyrax and althea. The joints were rubbed with volatile foap, and the patient was ordered to take a fpoonful of a draught compofed of one ounce of fyrup of caryoph. and fix grains of ammoniac, in fix ounces of water, and in the intervals an infufion of balm and fyrup of orange flowers was ordered. After ufing thefe remedies for feveral days, the fever abated, and ten grains of carbonat of potafs were added to each pint of barley water, of which the patient drank three chopins in twenty-four hours.

hours. She was farther directed to apply clysters prepared with soap wort and half a drachm of the carbonat of soda, and on the twenty-fourth day to take a purgative. In the course of five weeks she was completely cured, and recovered the use of her limbs.

---

The salutary effects resulting from the use of the carbonat of potass in puerperal fevers, appear to be sufficiently proved.

In the year 1771, M. Tissot, in his Essay on the diseases of people in general, proposed as a remedy for metastases of the milk, the oil of tartar (the carbonat of potass in a liquid state,) in doses of from twelve to fifteen or even twenty drops, three or four times a day, to be taken in a small quantity of water, broth, or gruel; a remedy from which he frequently observed the best effects to result. Van Stichel, a physician of Brusselles, in a small work entitled "Reflections on the acute diseases of women in child-bed, their nature, causes, and treatment in the Austrian Netherlands," printed in 1789, and which deserves to be more generally known, when speaking of this medicine, which he had adopted on the authority of Tissot, expresses himself in the following terms:

"Although I have coincided in opinion with the greater number of practitioners, with respect to the variety of acute diseases of women in child-bed, I have not been successful in their treatment. But since I am convinced that neither inflammatory nor putrid fevers, nor nervous affections, exist on such occasions, and being well persuaded that every acute fever which intervenes in women recently delivered is primarily induced by a fullness of the milk, or at least that the fever is complicated with this cause, and consequently ought to demand the principal attention of the physician, I have been more successful in my treatment of such diseases."

With an intention to correct the acescence of the diffused milk, and to prevent its ultimate coagulation, Van Stichel prescribed the purified oil of tartar per deliquium, from one to three drachms daily, in four ounces of distilled elder water, sweetened with two ounces of honey, to be taken by a table spoonful, either alone, or mixed in a pint of strong infusion of elder flowers, and two or three ounces of honey.

With this remedy alone, after he had, according to his usual practice, excited abundant perspiration, and encouraged it for several days, he often cured the most violent puerperal fevers.

"Others," adds the author, "have met with the same success by similar means, among whom is M. Van der Belen, Doctor of Medicine in the University of Louvain, who, in a letter dated October 3, 1787, expresses himself on this subject in in the following words: "I have succeeded so well with your alcaline mixture in puerperal fever, that I can easily dispense with any other remedy, and I need not even have recourse to ipecacuanha, whatever praise it may otherwise deserve."

Cit. Allan, our colleague, has several times employed this remedy: I have myself had occasion to apply it with advantage, and we have observed the best effects from the carbonat of pot-ass combined with bark, when the fever was of the remittent kind. This combination would be particularly useful in complicated cases of the puerperal with that of the gaol fever.

In extolling the method proposed by Cit. Guinot, we are far from wishing to depreciate the merit of that we have derived from the celebrated Doucet. But whatever success the latter may have been attended with, it can only be depended on when ipecacuanha has been given on the first attack of the disease, and even then it has sometimes proved ineffectual. Hence it is of advantage to introduce into medical practice another remedy, the efficacy of which is confirmed by a great number of experiments, and which may, in all cases, be usefully combined with that proposed by Doucet, as it will prove of great service when the latter has not been timely administered, or when it has failed to produce the desired effects.

---

### *Chronic Dysphagia, which originated from a ravenous Appetite. By* DR. HAGSTROM.

[From the New Transactions of the Royal Academy of Sciences at Stockholm. Vol. XIX. 1798.]

In the year 1797, a gardener died suddenly in the 62d year of his age, in consequence of a violent colic. This man was of a spare habit and thin appearance; he had always been afflicted with such a ravenous appetite, that he consumed as much food as four other persons, and nevertheless complained of insatiable hunger. He had frequently been subject to the colic and a violent heartburn, and three or four years previous to his death, he was confined to his bed for six months by a similar disease, attended with constant vomiting. For several years towards the latter period of his life he could take only liquid food, administered by spoonfuls. Dr. Hagstrom attended him for three years; but without being able to perform a cure. On dissecting the body after death, the lungs, liver and heart, were in a perfectly sound state; but the stomach was of a monstrous appearance, being three times the usual size. It contained from three to four quarts of matter resembling the yolk of an egg, when boiled hard and cut into small pieces, while it was extremely pungent and acid. In the mesentery were two small indurations, scarcely the size of a common pea. One part of the the œfophagus was fo indurated and conftricted, that a goofe quill could with difficulty be introduced. The parietes of this callous cylinder were half an inch thick, and its length meafured about two inches. The author is of opinion, that the diftention of the ftomach and its atonic ftate originated from the uncommon voracioufnefs of the patient; and there not being a fufficient quantity of the gaftric juice to diffolve the food, the ingefta had affumed fuch corrofive properties as readily produced the colic and heartburn; particularly as the œfophagus had been much ulcerated by frequent vomiting.

---

### *Some Obfervations on the dangerous Effect of cutting the Hair during the Convalefcence from Acute Difeafes.* By Cit. Lanoix.

[From the Bulletin of the Philomathic Society, No. 1, Germinal, 5th year of the Republic. April, 1797.]

The Author of this Memoir informs us, that if any natural emunctories are formed on the hairy part of the head, towards the decline of flow nervous fevers, it is of the greateft importance to preferve thefe canals, and particularly not to cut the hair, which defends thefe parts from the injurious accefs of the air.

Two women, in a very convalefcent ftate, whofe hair had been cut on their recovery from a putrid malignant fever, died foon after this imprudent action. A third owed her prefervation to her youth and the energy of her conftitution.

Cit. Lanoix has added to thefe facts fome reflections, by which he endeavours to prove, that when the cutting of the hair is attended with fatal confequences, they muft be afcribed to this circumftance, that the crifis directed by Nature towards the head, is impeded, and, as it were, arrefted in its progrefs. Confidering the hair as proper organs of fecretion which are fympathetically connected with the brain, and that they do not poffefs the property of conducting caloric, he concludes that they muft be important parts in promoting the crifis in difeafes, and that confequently they ought to be preferved; becaufe Nature fhould rather be affifted than difturbed in the meafures fhe adopts, with refpect to an organ fo effential to the procefs of life.

---

### *On the Effect of the Extract of Belladonna, applied to the Eyes previous to their being operated for the Cataract.*

Dr. Reimarus, of Hamburgh, a correfponding member of the Philomathic Society, has obferved, that on pouring into the eye a few drops of the extract of belladonna, diffolved in water, a temporary paralyfis took place, during which the pupil was uncommonly dilated, infomuch that the iris became almoft moft invifible. In confequence of this remarkable phenomenon, he has propofed this remedy with a view to prepare the eye for the operation of the cataract.

Dr. Grassmeyer, a fuccefsful oculift at Hamburgh, has actually employed this expedient in his practice, with uncommon advantage. The above-mentioned effect on the eye is produced within half an hour after the application of the remedy, fo that by the extreme dilatation of the pupil, the practitioner was enabled to commence the operation at the cornea, and proceed to the capfule of the cryftalline lens, without fear of hurting the iris. In fhort, the paralyfis effected on the retina, prevents the dreadful confequences which a fudden admiffion of light might probably occafion. *Ibid.* No. 3, (5th year).

---

*Extract from a Memoir by Cit. Sabatier, Profeffor at the School of Medicine in Paris, relative to the Amputation of the Arm at the Articulation.*

In later times many improvements have been made refpecting the method, as well as the means, employed in the amputation of the joint. Ingenious inftruments have been invented by the moft celebrated practitioners, for ftopping the violent flux of blood in amputation, and preventing hæmorrhages, which generally prove fatal. This operation is even at prefent allowed to be fo dangerous, that it ought never to be performed except in the moft defperate cafes. Some difeafed affections of the os humeri, and certain fractures caufed by gun-fhot wounds, oblige us, however, frequently to have recourfe to the inftrument. In the prefent memoir, Cit. Sabatier propofes to fubftitute another operation, which, by preferving the limb, and reftoring its power of motion, does not expofe the life of the patient to fo much danger. He then endeavours to prove, in four obfervations, that this operation is practicable, and that it is often partly accomplifhed by Nature alone. The firft of thefe obfervations is inferted in the 2d volume of the *Académie de Chirurgie*, by Boucher, who has extracted feveral pieces of bone from the articulation of the fcapula and humerus. The fecond obfervation, by Cit. Thomas, furgeon at Pézenas, is the following: A little girl, four years of age, after recovering from the confluent fmall-pox, was afflicted with an abfcefs, which opened fpontaneoufly, and ejected a portion of the os humeri, of the length of 0,04, without the periofteum, and deprived of the epiphyfis that formed its articulary head. It feparated after thirty days, without the aid of art. Cit. Thomas then began to extract the portion belonging to the articulation, and the wound wound was healed in the courfe of a month. The arm was not fenfibly diminifhed in length; and this patient, when fifteen years of age, had fo far recovered the ufe of it, that fhe was enabled to continue her ufual occupations as a fervant. Some time after fhe had the misfortune to be drowned, and a variety of circumftances intervened, which prevented Cit. Thomas from learning what change the bone had undergone.

A third obfervation, very fimilar to that above related, was communicated to the London Medical Society, and afterwards publifhed in a feparate work, under the title of "Chirurgical Obfervations," by Mr. White, of Manchefter. But in this cafe the effect of Nature was not waited for. The humerus was amputated a fecond time. Four months after, the patient was difcharged from the hofpital, cured: his arm was not diminifhed above 0,03; its form was unaltered, and he ufed it with as much ftrength and agility as that which had not been injured.

The fourth obfervation is by Cit. Vigaroux, furgeon at Montpellier. It is recorded in a memoir prefented to the Academy of Chirurgery, in the year 1774. The operation which this furgeon performed is of the fame nature as that of Mr. White; but it was performed too late, and the patient died in confequence of a metaftafis.

In thefe different obfervations, where the head of the humerus was detached from the corpus offis in confequence of difeafe, and where there was luxation, the amputation was eafily performed; but in a cafe of caries or exoftofis, which may require an amputation of the arm at the joint, this operation becomes more difficult, and requires other modes of proceeding. The following is the refult of Cit. Sabatier's method of operating, after a great number of trials on dead bodies.

The patient being feated on a chair, two incifions are made on the anterior and fuperior part of the arm, each 0,10 long, at the diftance of 0,05 longitudinally, and united below in the fhape of a V. The integuments, and that part of the deltoides mufcle, which fhould be comprehended in the incifion, being removed, the elbow muft be drawn behind, after which all the tendons of the mufcles fhould be cautioufly cut at the circumference of the capfule, together with three-fourths of the anterior parts of that membrane. The head of the bone fhould then be expelled through the wound, by cutting the attachments of the pectoralis major, the rotundus major, and the dorfalis magnus. The amputation of the bone muft be compleated, by paffing behind a thin pafteboard, yet fufficiently ftrong to guard the mufcles from the

 action action of the saw. An able assistant should place his finger upon the small arteries, to prevent too great a loss of blood. For the sake of precaution, another assistant should compress the humeral artery, by means of the instrument invented by Camper, viz. by supporting a thick compress between the coracoid process, the humeral extremity of the clavicle and pectoralis minor, or by another apparatus, such as that proposed in one of the French Journals of Medicine for the year 1765. The patient having been dressed after the operation, must be put to bed.

Cit. Sabatier concludes this memoir, by relating a cure, performed in consequence of a treatment analogous to what he proposes, and which he has since observed in the 64th volume of the Philosophical Transactions, by JACOB BENCK, surgeon, at Newcastle. *

" We may here be permitted," add the Editors of the Bulletin, " to take notice of the results of experiments on the same subject performed upon dogs, at the School of Medicine, by Cit. Chaussier, and which he has announced in his public lectures. The whole head of the femur was removed by the saw. The regenerated part of the bone recovered its rotatory motion, over the hip bones, although the limb remained a little shorter. This operation was not accompanied with any distressing symptoms. *Ibid.* No. XXXIV. *Nivose*, 8th year.

---

### *On the Chemical Analysis of Vegetables. By Dr.* HERMBSTÆDT, *of Berlin.*†

[ Continued from our first Volume, p. 506. ]

### *On the Separation of Mucilage from Vegetable Bodies.*

Pure mucilaginous matter is found in very few vegetables, in a separate state, as it is usually intermingled with gum. In order to separate both ingredients carefully from each other, the plant submitted to analysis must be treated precisely according to the method already pointed out on a former occasion; ‡ the dry mass thus obtained,§ should previously be weighed with great

---

* We have not been able to find this Case in the volume here mentioned. EDIT.

† We have at length received the continuation of these valuable papers, which we are happy to lay before our readers, without farther delay, as we are in possession of the whole. EDIT.

‡ Vide the process for separating gum, as stated in our Journal, Vol. I. pp. 504, and fol. § Ibid. p. 505.—8.

great accuracy, and then diſſolved in the ſmalleſt poſſible quantity of pure water. Into this ſolution, vitriolic acid diluted with an equal quantity of diſtilled water ſhould be dropped by ſmall portions: by theſe means the mucilage will ſoon be reduced to a coagulated ſtate, while the matter of gum will remain unchanged. When no farther coagulation takes place, the whole maſs is ſuffered to ſtand at reſt, that the ſeparated mucilage may depoſit itſelf on the bottom of the veſſel, where it acquires a gelatinous conſiſtence. The ſupernatant clear fluid ſhould then be carefully decanted, but the mucilaginous reſiduum muſt be evaporated to dryneſs by a gentle heat, till it becomes of a conſiſtence ſimilar to horn.—To determine the relative proportion of mucilaginous matter exiſting in a certain vegetable, there is nothing farther required than to deduct the loſs which the whole maſs has ſuſtained, by balancing it againſt the actual weight of this horny reſiduum.

*On the Separation of Albumen from Vegetables.*

As the albumen of dried vegetables acquires ſuch a degree of hardneſs, that it can neither be diſſolved nor ſoftened by water, it is an almoſt indiſpenſable requiſite to chooſe the vegetables in a freſh and ſucculent ſtate, in order to effect its ſeparation. The various ſpecies of grain however, and fruits of the pulſe kind, make an exception to this rule; becauſe in theſe, the albumen is protected againſt complete exſiccation, by its intermixture with other conſtituent parts: in theſe alſo the ſeparation of the albumen muſt be accompliſhed by a very different proceſs.

We ſhall firſt point out the method of analyzing freſh and ſucculent vegetables, with which the operator proceeds in the following manner:

1. A determinate quantity of freſh plants is accurately weighed, then bruiſed in a ſtone mortar, and the ſap properly expreſſed. The woody reſiduum is macerated in cold water, the whole maſs well ſtirred, and the fluid part again expreſſed and mixed with the former. The whole liquor is then expoſed to reſt for ſeveral hours, that the fibres in the other foreign particles may fall to the bottom; a clear fluid is then decanted, which contains the albumen mixed with other ingredients.

2. The whole liquor is next poured into a tin kettle, or a glaſs retort, and expoſed to a heat of 70 degrees of De Luc, or 190 degrees of Fahrenheit. In this temperature, all the albumen coagulates, and appears on the ſurface of the fluid, like a connected plaſtic matter.

3. After the whole has been ſuffered to grow cold, the remaining liquor at length becomes perfectly clear. It is then filtered through blotting paper previouſly weighed, and the albumen left on the paper is purified from the adhering foreign particles, by frequent affuſions of cold water; it is afterwards dried, and its weight determined by the ſcale.

By this method we may diſcover the proportion of albumen in a freſh plant; but in order to aſcertain this proportion likewiſe in a dry plant, it is only neceſſary to compare the relative weight of vegetables in a freſh and dried ſtate; thus, for inſtance, if eight ounces weight of a freſh plant ſhould produce 120 grains of albumen, and if eight ounces of a freſh plant ſhould be equal to two ounces in a dried ſtate, it follows that one pound of the dry plant will contain 960 grains of albumen.

### *On the Separation of the Albumen from the various Species of Grain and the leguminous Seeds.*

To determine the quantity of albumen contained in various kinds of grain, and the leguminous ſeeds, the following proceſs ought to be adopted:

The ſubſtance under experiment is firſt reduced to the fineſt powder, a certain quantity of it is weighed, and mixed into a ſolid paſte with cold water; which paſte is then tied up in a cloſe piece of linen, and immerſed in cold water, where it is kneaded with the hand, till the repeated affuſions become no longer turbid, but remain perfectly clear.

By this ſimple proceſs the farinaceous ingredients, as well as the gummy and ſaccharine particles, combine with the water, while the albumen remains on the linen, and exhibits a light grey, tough, and elaſtic ſubſtance. In this ſtate it ſhould be perfectly dried, and its weight properly aſcertained.

### *On the Separation of the Caoutchouc, commonly called Gum Elaſtic.*

This elaſtic matter is a conſtituent part of various vegetable productions, though it is of all gum-reſinous ingredients the moſt difficult of ſeparation. If it be intermingled with ſome of the reſinous ſubſtances, ſuch as the gum maſtich, it may thence be obtained tolerably pure, by a previous digeſtion and ſeparation of the reſinous part with the aid of rectified ſpirit of wine. It may be ſeparated with equal facility from the different ſpecies of miſſeltoe,* by waſhing it in water, with which

* M. Thielebein, a German chemiſt, has firſt proved, in "Crell's *Neueſten Entdeckungen der Chemie,*" Part VII. p. 58, that the glutinous part of the miſſeltoe is perfectly analogous to the elaſtic gum; a fact which he has confirmed by experiments.—Is it not probable that this ſubſtance, in general, conſiſts of a combination of vegetable mucilage with the aſtringent principle, or matter?—The reader is requeſted to compare this problem with the ſubſequent obſervations relative to the ſeparation of the aſtringent matter—in our next.

which it readily combines. But the proceſs of extracting the caoutchouc is attended with much greater difficulties in thoſe vegetables where it is mixed with gummy and ſaponaceous ingredients; yet here likewiſe the operator will ſucceed in the attempt, if he proceeds in the following manner:

1. A certain quantity of a plant aſcertained by weight, is repeatedly digeſted in alkohol and water, till the fibrous and woody reſiduum imparts neither taſte nor colour to the mixture: theſe ſolvents deprive the vegetables, under experiment, of every other ingredient, except the caoutchouc, which remains undiſſolved.

2. The reſiduum, after being dried, ought to be ſtrongly digeſted with four or ſix parts of rectified petroleum, which diſſolves the elaſtic gum, and leaves the fibrous part of the vegetable at the bottom of the veſſel.

3. The liquor obtained by expreſſing the whole through linen cloth, ſhould be left to ſettle for ſeveral days, after which the clear fluid is poured off, and mixed with a third part of water; this mixture ought to be diſtilled over in a retort, where the caoutchouc matter remains in the reſiduum, forming a tough, elaſtic maſs—the weight of which may be determined after it has been completely dried.

### *On the Separation of Wax from Vegetables.*

If we are induced to believe, either from external appearances, or more correctly from a previous examination, inſtituted according to the directions formerly given,* that a vegetable body contains matter of wax, we may adopt for its ſeparation the following moſt convenient method:

1. The ſubſtances under experiment muſt be previouſly freed from all other ingredients which are ſoluble in water and alkohol; the reſiduum is then mixed, either with ſix times its weight of cauſtic ammonia, or ſtrongly digeſted in a weak lixivium of cauſtic-natron. Theſe fluids combine with the matter of wax, and render it ſoluble in its preſent watery medium.

2. The liquor thus obtained is carefully cleared from the reſiduum, then filtered, and afterwards a diluted ſulphuric acid is dropped into it, while it is inceſſantly ſtirred, till the acid predominates in the mixture. The matter of wax, which by this treatment is ſeparated in the form of a pale yellow powder, ſhould be completely edulcorated in water, and melted over a gentle fire: it will now appear in its pure form, ſo that its weight may be determined accordingly.

[ To be continued in our next Number. ]

* See the firſt Volume of this Journal, p. 268. H.

